# Pathobiology of Tennessee 2017 H7N9 low and high pathogenicity avian influenza viruses in commercial broiler breeders and specific pathogen free layer chickens

**DOI:** 10.1186/s13567-018-0576-0

**Published:** 2018-08-29

**Authors:** Kateri Bertran, Dong-Hun Lee, Miria F. Criado, Diane Smith, David E. Swayne, Mary J. Pantin-Jackwood

**Affiliations:** Exotic and Emerging Avian Viral Diseases Research Unit, Southeast Poultry Research Laboratory, U.S. National Poultry Research Center, Agricultural Research Service, U.S. Department of Agriculture, 934 College Station Rd, Athens, GA 30605 USA

## Abstract

In March 2017, H7N9 highly pathogenic avian influenza (HPAI) virus was detected in 2 broiler breeder farms in the state of Tennessee, USA. Subsequent surveillance detected the low pathogenicity avian influenza (LPAI) virus precursor in multiple broiler breeder farms and backyard poultry in Tennessee and neighboring states. The pathogenesis of the H7N9 LPAI virus was investigated in commercial broiler breeders, the bird type mostly affected in this outbreak. Infectivity, transmissibility, and pathogenesis of the H7N9 HPAI and LPAI viruses were also studied in 4-week-old specific pathogen free (SPF) leghorn chickens. The mean bird infectious doses (BID_50_) for the LPAI isolate was 5.6 log_10_ mean egg infectious dose (EID_50_) for broiler breeders and 4.3 log_10_ EID_50_ for SPF layer chickens, and no transmission to contact-exposed birds was observed. In both bird types, virus shedding was almost exclusively from the oropharyngeal route. These findings suggest sub-optimal adaptation for sustained transmission with the H7N9 LPAI isolate, indicating that factors other than the birds genetic background may explain the epidemiology of the outbreak. The BID_50_ for the HPAI isolate in SPF layer chickens was more than 2 logs lower (<2 log_10_ EID_50_) than the LPAI isolate. Also, the HPAI virus was shed by both the oropharyngeal and cloacal routes and transmitted to contacts. Greater susceptibility and easier transmission of the H7N9 HPAI virus are features of the HP phenotype that could favor the spread of HPAI over LPAI viruses during outbreaks.

**Electronic supplementary material:**

The online version of this article (10.1186/s13567-018-0576-0) contains supplementary material, which is available to authorized users.

## Introduction

Waterfowl are the natural reservoirs of avian influenza (AI) virus and usually carry the low pathogenic (LP) phenotype [1–3]. Occasionally, LPAI viruses can transmit from wild birds to domestic poultry resulting in subclinical infections or mild respiratory disease and drops in egg production, although adverse conditions can predispose birds to a more severe disease [4]. After circulating in gallinaceous poultry, some H5 and H7 LPAI viruses can mutate to the highly pathogenic (HP) phenotype, causing severe systemic disease and high mortality in domestic birds [5]. The acquisition of multiple basic amino acids at the cleavage site of the hemagglutinin (HA) via recombination, insertion, or mutations is a molecular determinant for high pathogenicity. Consequences of AI virus infection in poultry include negative economic impact on agriculture and a potential source for pandemic viruses in humans [6, 7].

In March 2017, concurrent outbreaks of H7N9 LPAI and HPAI virus were confirmed in broiler breeder flocks in the state of Tennessee, USA, with additional commercial broiler breeder flocks and mixed species in backyard flocks within the states of Alabama, Kentucky, and Georgia being affected with H7N9 LPAI virus [8, 9]. Surveillance data and genetic analyses suggested multiple introductions of LPAI virus before mutation to high pathogenicity and inter-farm transmission [8]. Phylogenetic analyses based on complete genome sequences also showed that these viruses were closely related to a wild duck-origin H7N9 LPAI virus isolated 6 months previously in Wyoming [8]. The inserted sequences at the HA cleavage site (PENPKTDRKSRHRRIR/G, insertion sequence is underlined) in H7 viruses had 100% sequence homology to chicken 28S rRNA, suggesting that the mutation occurred during virus replication in chickens [8]. Therefore, a LPAI virus was likely transmitted from wild aquatic birds to poultry and subsequently mutated to HPAI virus within one broiler breeder flock before spreading to a second broiler breeder flock within the control zone. Fortunately, the outbreak was effectively controlled in a timely fashion, with no further premises affected and no human infections identified.

Different genetic background between layer- and broiler-type chickens may have an effect not only on performance but also on genetic expression and immunological responses [10–12], accounting for differences in susceptibility to AI virus infection between both chicken types [13–20]. We recently showed that broilers, regardless of age, were less susceptible to H5N2 HPAI virus (Midwestern USA, 2015) than layers, but similarly susceptible to turkeys [21–23]. Since this H5N2 HPAI virus outbreak (Midwestern USA, 2015) affected commercial turkey and layer farms but not broiler farms [24], our findings suggested that genetic resistance of broilers to infection may have partially accounted for the lack of affected broiler premises, but other factors such as fewer outside-to-on-farm exposure to contacts, type of production management system, leaving broiler farms unpopulated in the control zone, or enhanced biosecurity, could have resulted in the lack of broiler farms being affected [21]. Different from recent HPAI virus outbreaks in poultry in the USA, i.e. H5N2 in Midwest and H7N8 in Indiana, which involved mostly turkeys and layers [24, 25], the Tennessee 2017 H7N9 AI virus outbreaks affected mainly broiler breeder commercial premises [8, 9]. The absence of affected layer premises could be due to their low number in the affected region or failure to introduce a H7N9 virus onto the farms due to management factors [9].

Understanding breed-related differences in susceptibility to AI virus infections can help elucidate the complex pathobiology of AI and decisively impact optimal management of outbreak control strategies. In the present study, pathogenesis of the Tennessee 2017 H7N9 LPAI virus was investigated in commercial broiler breeders, the bird type mostly affected in this outbreak. In order to compare the infectivity, transmissibility, and pathogenicity of the Tennessee 2017 H7N9 LPAI and HPAI isolates with AI viruses from similar previous outbreaks, such as H7N8 HPAI virus (Indiana, 2016), we also studied the viruses in young SPF layer chickens.

## Materials and methods

### Viruses

Egg passage stocks of A/chicken/Tennessee/17-007431-3/2017 H7N9 LPAI virus (GenBank accession numbers KY818816-KY818823) and A/chicken/Tennessee/17-007147-2/2017 H7N9 HPAI virus (GenBank accession numbers KY818809–KY818815) were provided by the National Veterinary Services Laboratories, Animal and Plant Health Inspection Service (APHIS), United States Department of Agriculture (USDA). The H7N9 LPAI and HPAI isolates differ by a 9-amino-acid insertion in the HA gene and 18 additional amino acids throughout the genome [8]. Working stocks were prepared (egg passage 1) and titrated in embryonating chickens eggs (ECE) using standard methods [26]. Stocks were diluted to the target dose with brain heart infusion (BHI) broth (Becton, Dickinson and Company, Sparks, MD, USA). The studies were performed in biosecurity level-3 enhanced (BSL-3E) facilities in accordance with procedures approved by the Institutional Biosafety Committee of the U.S. National Poultry Research Center (USNPRC), Agricultural Research Service (ARS), USDA.

### Animals and housing

Cobb broiler breeders in lay were obtained from a commercial producer (courtesy of John Smith and Sarah Tilley, Fieldale Farms Corp., Baldwin, GA, USA). Specific pathogen free (SPF) White Leghorn chickens were obtained from the USNPRC in-house flocks. At 4 weeks of age (SPF layer chickens) or 36 weeks of age (broiler breeders), birds were transferred to animal BSL-3E facilities at the USNPRC for challenge. Representative number of each bird type was bled immediately prior to challenge to confirm the absence of AI virus antibody by hemagglutinin inhibition (HI) assays. Each experimental group was housed in self-contained isolation units ventilated under negative pressure with inlet and exhaust HEPA-filtered air. The birds had ad libitum access to feed and water. This study was reviewed and approved by the USNPRC Institutional Animal Care and Use Committee. Assessment of egg production in broiler breeders and virus contamination of eggs was not possible because housing in isolation cabinets prevented collection of eggs.

### Experimental design and sampling

#### Infectivity and transmission

To evaluate the mean bird infectious dose (BID_50_) and lethal dose (BLD_50_) of the viruses, birds were divided into groups as shown in Table 1 and individually tagged for identification. The inocula were prepared by diluting working virus stocks to approximately 2 (low dose), 4 (medium dose), or 6 (high dose) log_10_ mean egg infectious doses (EID_50_) in 0.1 mL and were administered by the intra-choanal route to 5 birds per dose. The inocula titers were subsequently verified by back titration in ECE as within 0.5 log_10_ of the target titer for all groups. In addition, 5 sham-exposed birds from each bird type were inoculated with 0.1 mL of sterile allantoic fluid diluted 1:300 in BHI media. In SPF layer chickens, contact-exposure transmission was evaluated by adding 3 non-inoculated hatch-mates (contacts) to each dose group at 1 day post-challenge (dpc). Clinical signs were monitored daily. Oropharyngeal (OP) and cloacal (CL) swabs were collected from all birds at 2, 4, 7, and 10 dpc, placed in 1.5 mL of BHI with penicillin (2000 units/mL; Sigma Aldrich, St. Louis, MO, USA), gentamicin (200 μg/mL; Sigma Aldrich) and amphotericin B (5 μg/mL; Sigma Aldrich), and stored at −80 °C until use. Severely sick birds were euthanized and counted as dead for the next day in mean death time (MDT) calculations. At 14 dpc, survivors were bled to evaluate seroconversion and euthanized.

**Table 1:** **Infectivity, lethality, and transmission study design and summary results**

Bird type (age)	Challenge virus	Dose (log_10_)	Inoculated infected/total^a^	BID_50_ (log_10_)	Inoculated dead/total (MDT^b^)	BLD_50_ (log_10_)	Contact-exposed infected/total^c^
Broiler breeders (36w)	H7N9 LPAI	2	0/5	5.6	0/5	>6	nd
		4	2/5		0/5		nd
		6	2/5		0/5		nd
	–	sham	0/5	–	0/5	–	–
SPF White Leghorn (4w)	H7N9 LPAI	2	0/5	4.3	0/5	>6	0/3
		4	2/5		0/5		0/3
		6	5/5		0/5		0/3
SPF White Leghorn (4w)	H7N9 HPAI	2	3/5	<2	3/5 (2.3)	<2	0/3
		4	5/5		5/5 (≤2.4)		0/3
		6	5/5		5/5 (2.2)		1/3 (dead)
	–	sham	0/5	–	0/5	–	–

BID_50_: mean bird infectious dose, BLD_50_: mean bird lethal dose, MDT: mean death time in days, nd: not determined.

^a^Birds were considered infected if they shed virus and/or were positive for antibodies at 14 dpc.

^b^#dead birds × dpc/total dead birds.

^c^Contact-exposed birds were considered infected if they died, shed virus, and/or were positive for antibodies at 14 dpc.

#### Pathogenesis

To evaluate pathogenicity, 3 broiler breeders were challenged with 6 log_10_ EID_50_ of the H7N9 LPAI virus, 10 SPF layer chickens were challenged with 6 log_10_ EID_50_ of the H7N9 LPAI virus, and 10 SPF layer chickens were challenged with 6 log_10_ EID_50_ of the H7N9 HPAI virus. At 2 dpc (for HPAI group) or 3 dpc (for LPAI groups), 2 SPF layers and 3 broiler breeders per group were euthanized for necropsy to examine for gross lesions and collect tissues for microscopic evaluation. Two sham-exposed birds per group were also euthanized and necropsied as control birds. A full set of tissues was collected from each bird and fixed in 10% neutral buffered formalin solution (Thermo Fisher Scientific, Waltham, MA, USA), paraffin-embedded, sectioned, and stained with hematoxylin-and-eosin (HE). Duplicate sections were stained by immunohistochemistry (IHC) to visualize the distribution of influenza virus antigen in individual tissues using a mouse-derived monoclonal antibody (P13C11, developed at SEPRL) specific for type A influenza virus nucleoprotein [27]. Lung, spleen, heart, muscle, and brain (SPF layer chickens and broiler breeders), as well as portions of each section of the reproductive tract, i.e. ovary, infundibulum, magnum, isthmus, and shell gland (broiler breeders) were also collected and frozen at −80 °C for subsequent virus detection.

#### Viral RNA quantification in swabs and tissues

Swabs and tissues were processed for quantitative real-time RT-PCR (qRRT-PCR) to determine total viral RNA. Virus titers in tissue samples were determined after weighing, homogenizing, and diluting tissues in BHI to a 10% (wt/vol) concentration. Total RNA was extracted from tissues using Trizol LS reagent (Invitrogen, Carlsbad, CA, USA) and the Qiagen RNeasy Mini Kit (Qiagen Corp, Valencia, CA, USA) was used to recover RNA from the aqueous phase. Equal amounts of RNA extracted from the tissue samples were used in the qRRT-PCR assay (50 ng/μL). Total RNA was extracted from swabs using MagMAX™-96 AI/ND Viral RNA Isolation Kit^®^ (Ambion, Inc., Waltham, MA, USA). The resulting tissue and swab viral RNA extracts were quantified by one-step qRRT-PCR targeting the influenza matrix gene [28] using 7500 FAST Real-time PCR System (Applied Biosystems, Foster City, CA, USA). For virus quantification, standard curves were established with RNA from dilutions of the egg passage 2 of the challenge viruses (no significant differences in matrix detection were observed between egg passages 1 and 2). Results were reported as EID_50_/mL or EID_50_/g equivalents. The lower detection limit for H7N9 LPAI virus was 0.9 log_10_ EID_50_/mL (1.9 log_10_ EID_50_/g for tissue samples). The lower detection limit for H7N9 HPAI virus was 1.5 log_10_ EID_50_/mL (2.5 log_10_ EID_50_/g for tissue samples). For statistical purposes, qRRT-PCR negative swabs were given the value of 0.1 log_10_ below the corresponding qRRT-PCR test limit of detection. Significant difference for mean viral titers between groups was analyzed using Mann–Whitney test (GraphPad Prism™ Version 5 software). A *p*-value of < 0.05 was considered to be significant.

#### Serology

Sera samples were tested by HI assays [29] against challenge virus antigens [30]. Titers were calculated as the reciprocal of the last HI positive serum dilution and samples with HI titers of 8 (2^3^) or below were considered negative. Sera samples were also tested using a commercial ELISA (AI Multi-S-Screen, IDEXX, Westbrook, ME, USA).

## Results

### Infectivity, transmission, and pathogenicity of the H7N9 LPAI virus in broiler breeders

**Figure 1:**
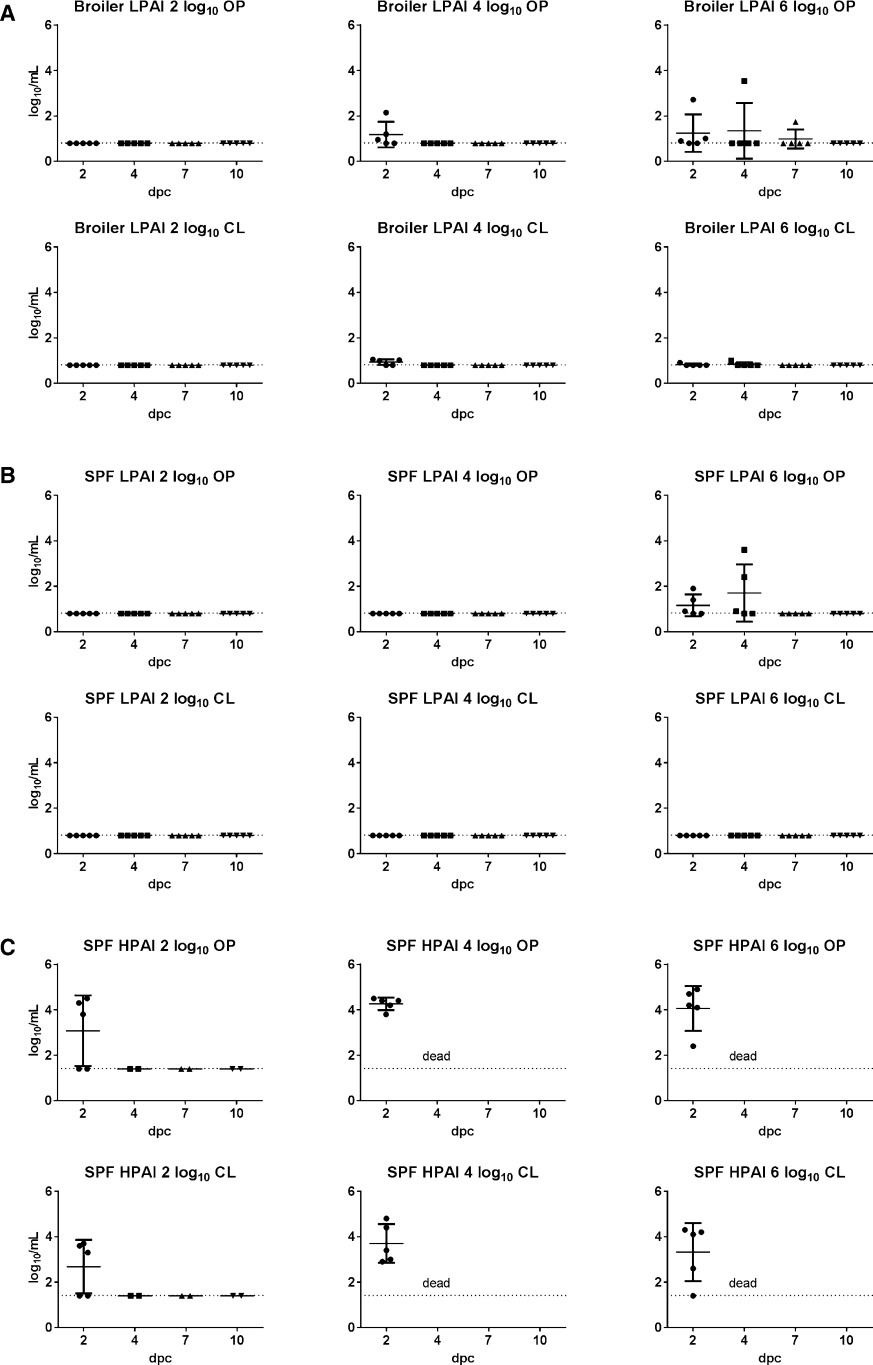
**Scatter plot of oropharyngeal (OP) and cloacal (CL) virus shedding. A** Broiler breeders inoculated with H7N9 LPAI virus. **B** SPF White Leghorn chickens inoculated with H7N9 LPAI virus. **C** SPF White Leghorn chickens inoculated with H7N9 HPAI virus. Virus detection by qRRT-PCR. Shedding titers are expressed as log_10_ with error bars. For statistical purposes, negative samples were given the value of 0.1 log_10_ below the qRRT-PCR test limit of detection (0.8 log_10_ EID_50_/mL for H7N9 LPAI virus and 1.4 log_10_ EID_50_/mL for H7N9 HPAI virus).

Based on serology and viral RNA detection in swabs, no broiler breeders were infected in the lowest dose group, while 40% were infected in the medium and high dose groups; the resulting BID_50_ was 5.6 log_10_ EID_50_ (Table 1). Virus was only detected at 2 dpc in OP swabs of 2/5 chickens inoculated with the medium dose, and up to 7 dpc in OP swabs of 1/5 chickens inoculated with the high dose (Figure 1A). Virus in CL swabs was not detected or detected at minimal titers in the medium and high dose groups. The LPAI virus did not cause clinical disease in infected broiler breeders, and no gross lesions or histopathological findings were observed in the 3 chickens necropsied at 3 dpc from the high dose group. In addition, no virus titers were detected by qRRT-PCR in any of the tissues tested (Table 2).

**Table 2:** **Virus detection and titers in tissues from broiler breeders and SPF White Leghorn chickens inoculated with Tennessee 2017 H7N9 LPAI or HPAI viruses**

Bird type (age)	Pathotype	Day post-challenge	No. with virus detected/total (titer as log_10_ EID_50_/g)^d^					
			Lung	Spleen	Heart	Brain	Muscle	Reproductive tract^e^
Broiler breeders (36w)^a^	LP	3	0/3	0/3	0/3	0/3	0/3	0/3
SPF White Leghorn (4w)^b^	LP	3	0/2	0/2	0/2	0/2	0/2	nd
SPF White Leghorn (4w)^c^	HP	2	2/2 (7.2 ± 0.3)	2/2 (7.3 ± 0.2)	2/2 (8.3 ± 0.3)	2/2 (7.0 ± 0.6)	2/2 (7.5 ± 0.4)	nd

Virus detection by qRRT-PCR.

nd: not determined.

^a^Tissues from 3 broiler breeders necropsied at 3 dpc. The threshold of detection in tissues was 1.9 log_10_ EID_50_/g for H7N9 LPAI virus.

^b^Tissues from 2 SPF layer birds necropsied at 3 dpc. The threshold of detection in tissues was 1.9 log_10_ EID_50_/g for H7N9 LPAI virus.

^c^Tissues from 2 SPF layer birds necropsied at 2 dpc. The threshold of detection in tissues was 2.5 log_10_ EID_50_/g for H7N9 HPAI virus.

^d^Mean titer ± SD.

^e^All sections of the reproductive tract were tested: ovary, infundibulum, magnum, isthmus, and shell gland.

### Infectivity, transmission, and pathogenicity of the H7N9 LPAI virus in SPF layer chickens

Based on serology and virus RNA detection in swabs, no SPF layers were infected in the lowest dose group, while 40% and 100% were infected in the medium and high dose groups, respectively; the resulting BID_50_ was 4.3 log_10_ EID_50_ (Table 1). Virus was only detected at 2 and 4 dpc in OP swabs of 3/5 chickens inoculated with the highest dose, while virus in CL swabs was not detected in any dose group (Figure 1B). No contact chickens were infected in any of the dose groups based on lack of seroconversion and lack of virus detection in swabs (Table 1). The LPAI virus did not cause clinical disease in infected SPF layers, and no gross lesions were observed in the 2 chickens necropsied at 3 dpc from the pathogenesis group. Histologically, mild lymphocytic rhinitis and tracheitis were observed in both chickens examined and was associated with rare AI virus antigen staining in epithelial cells and infiltrating macrophages of the nasal cavity turbinates and trachea. No virus titers were detected by qRRT-PCR in tissues of the 2 necropsied birds (Table 2).

### Infectivity, transmission, and pathogenicity of the H7N9 HPAI virus in SPF layer chickens

Sixty percent mortality was observed in the lowest dose group, while 100% of the birds died in the medium and high dose groups (MDT 2.3 days); the resulting BLD_50_ was <2 log_10_ EID_50_ (Table 1). Survivors lacked clinical signs, virus shedding, and seroconversion, and thus were considered uninfected. In contrast, most birds that became infected and died or were euthanized shed a high quantity of virus prior to death, both via the oropharynx and the cloaca (Figure 1C). Also, virus titers shed via the OP route at peak shedding day were significantly higher in H7N9 HPAI group (mean 4.1 log_10_ EID_50_) than H7N9 LPAI groups (means 1.3 and 1.7 log_10_ EID_50_ for broiler breeders and SPF layers, respectively) (data not shown). The resulting BID_50_ was <2 log_10_ EID_50_ (Table 1). One contact bird of the high dose group died at 4 dpc. The majority of birds that died did so by 2 dpc without showing clinical signs (i.e. peracute disease). Birds that took a day or more to die were euthanized because of severe clinical signs including swollen heads, ruffled feathers, conjunctivitis, lethargy, anorexia, prostration, and cyanotic combs and wattles. The two necropsied birds had empty intestines and were dehydrated. Petechial hemorrhages were observed in the eyelid of one bird. Similar type and severity of histological lesions were observed in both birds examined (see Additional file 1). Moderate to severe, multifocal necrosis was present in the parenchymal cells of many tissues but especially in lung, heart, spleen, and adrenal gland, in some cases accompanied with mild to severe inflammation. Staining for viral antigen was present in areas of necrosis and infiltrating mononuclear cells in many tissues including lymphoid tissues, lung, brain, liver, adrenal gland, and spleen. Staining was also present in parenchymal cells of some organs, including cardiac myocytes, Kupffer cells, hepatocytes, microglial cells and neurons, epithelium of air capillaries in the lung, kidney tubular epithelial and glomerular cells, and feather follicle epithelial cells. Viral antigen was commonly detected in vascular endothelial cells in the nasal cavity, trachea, eyelid, and comb, while in all other tissues viral antigen was only detected in a few, individual vascular endothelial cells. High virus titers (7.0–8.3 log_10_ EID_50_/g) were detected by qRRT-PCR in brain, spleen, heart, lung, and muscle of both necropsied birds (Table 2).

## Discussion

In this study, neither broiler breeders nor SPF layer birds exhibited clinical signs or death when experimentally infected with the Tennessee 2017 H7N9 LPAI isolate. This is consistent with a report by Spackman et al. [31] where chickens showed high seroconversion rates (70–100%) to all tested North American H7 LPAI viruses (*n* = 12) following experimental inoculation studies, but exhibited sub-clinical to mild diseases. However, severe clinical disease has occurred with some LPAI viruses in the field when accompanied by concomitant factors [4]. In fact, in the currently investigated outbreak, some H7N9 LPAI virus-affected commercial broiler breeder barns had drops in egg production for a short period of time and increased mortality [9]. Although LPAI viruses are generally restricted to the respiratory and gastrointestinal tracts when not inoculated by intravenous or intramuscular injection [32, 33], some LPAI viruses have been isolated from a limited number of other tissues including the pancreas, kidneys, and oviduct of intranasally inoculated chickens [34, 35], and from the kidneys, ovary, and oviduct of intratracheally inoculated birds [4, 36]. In our study, no LPAI virus was detected in the reproductive tract or other tissues of the broiler breeders, indicating that other factors, infectious and non-infectious, might aggravate the clinical presentation of LPAI virus under field conditions.

The estimated BID_50_ for the LPAI isolate was 4.3 log_10_ EID_50_ for SPF layer chickens, versus 5.6 log_10_ EID_50_ for broiler breeders. While these BID_50_ were lower than BID_50_ for non-poultry origin North American H7 LPAI viruses (6.2–6.9 log_10_ EID_50_) [37], no contact-exposed SPF layer birds were infected in our study, suggesting insufficient chicken adaptation of Tennessee H7N9 LPAI viruses for sustained transmission. Although transmission was not evaluated in broiler breeders, similar virus shedding dynamics between both bird types and higher BID_50_ for broiler breeders suggest that transmission would have been unlikely in the broiler breeder groups. It is worth noting that neither broiler breeders nor SPF layers shed LPAI virus by the CL route, which lack of can critically hinder virus transmission among birds [38]. Field conditions, including associated secondary infections, immunosuppression, or adverse environmental conditions, might lower the minimum AI virus exposure dose needed to infect commercial broiler breeders, thus emphasizing the importance of good biosecurity and management practices in controlling AI virus, as previously suggested by our group [21]. As aforementioned, the absence of affected layer premises could be due to a low number of layer farms compared to broiler breeder farms in the affected region or failure to introduce AI virus onto the farms due to management factors [9]. Therefore, the two systems vary in conditions that could impact virus exposure and maintenance, as well as host susceptibility and detection [9].

The H7N9 HPAI isolate had better infectivity and transmissibility in SPF chickens than its LPAI precursor. The BID_50_ for the HPAI isolate was more than 2 logs lower (<2 log_10_ EID_50_) than the LPAI isolate (4.3 log_10_ EID_50_). In addition, H7N9 HPAI-infected groups showed replication and shedding pattern differences compared to LPAI-infected groups: significant higher virus shedding titers, higher (although not significant) number of birds shedding, and gastrointestinal replication detected via CL swabs in addition to respiratory tract replication detected via OP swabs. Consistent with our findings, higher infectivity, lethality, and transmissibility of the HPAI phenotype were demonstrated in chickens for the H7N8 (Indiana, 2016) and H5N2 HPAI (Pennsylvania, 1983) viruses compared to their LPAI phenotype precursor [39, 40]. Collectively, these differences likely contributed to an increased efficiency of HPAI viral replication and contamination in the environment, with subsequent better transmission to HPAI virus contact-exposed birds. Transmission efficiency in our experimental conditions is likely underestimated due to an artifact of our housing (isolators with high rates of airflow and grate floors) as discussed previously [40] and lack of concomitant management factors that might be encountered in commercial farms which can increase environmental contamination, individual bird exposure, and inter-farm transmission.

Before the H7N9 outbreak in Tennessee in 2017, the most recent H7 HPAI outbreak in USA occurred in 2016, affecting turkeys in Indiana [25]. Both the H7N9 Tennessee (2017) and H7N8 Indiana (2016) events were identified when a LPAI virus mutated to HPAI virus, and samples were collected in response to clinical signs observed in the birds [9]. The distribution of cases and the presence of antibody-positive flocks for the H7N9 2017 outbreak suggest that detection occurred later during the course of infection as compared to the H7N8 2016 outbreak, where infection was detected shortly after virus introduction based upon virus detection in turkeys in both HPAI and LPAI virus-affected barns [9]. During both the H7N9 2017 and the H7N8 2016 events, HPAI virus-infected flocks were rapidly detected, quarantined, and depopulated [9], leading to single-farm spread in H7N9 2017 outbreak and no farm-to-farm spread in H7N8 2016 outbreak [40]. Such prompt detection resulted in outbreaks of short duration by limiting environmental contamination and potential for virus spread to susceptible flocks regardless of the infectivity of the virus and the titers of virus shed [40]. The H7N9 LPAI and HPAI isolates tested here had similar BID_50_ in SPF layer chickens to the H7N8 virus (Indiana, 2016) [40]. Regarding genetic relatedness, the H7N9 2017 and H7N8 2016 viruses share five of eight genes (PB2, PB1, HA, NP, MP) but are clearly different in the other three genes (PA, NA, NS) [9]. All H7N9 viruses isolated from the 2017 outbreak shared high levels of nucleotide identity (>99.2–99.7%) across all 8 gene segments except for the insertion at the HA cleavage site in the HPAI viruses [8]. However, based on genetic analysis, more than one H7N9 virus introduction occurred from wild birds into commercial broiler breeders and backyard poultry, with the LPAI virus circulating undetected in poultry in the USA southeast region for 1–3 months [8]. Consequently, differences in infectivity, transmission, and pathogenicity among different LPAI virus isolates from this outbreak might exist.

In conclusion, we observed inadequate or sub-optimal adaptation for sustained transmission with the H7N9 LPAI isolate in both broiler breeders and SPF layer birds. These findings suggest that not only the bird genetic background but also other factors including the birds immunological status, field production conditions, biosecurity, and management practices are involved in the epidemiology of the outbreak which affected mainly broiler breeder commercial premises [8, 9]. In addition, higher susceptibility and transmissibility of the H7N9 HPAI virus are features of the HP phenotype that could help in the spread of HPAI viruses during outbreaks.

## Additional file

**Additional file 1.** **Microscopic lesions and viral antigen distribution in tissues from chickens inoculated with H7N9 HPAI virus.** Four week-old specific pathogen free (SPF) White Leghorn chickens were challenged with A/chicken/Tennessee/17-007147-2/2017 H7N9 HPAI virus and sampled at 2 dpc.

## Abbreviations

AI: avian influenza
APHIS: Animal and Plant Health Inspection Service
ARS: Agricultural Research Service
BHI: brain heart infusion
BID_50_: mean bird infectious dose
BLD_50_: mean bird lethal dose
BSL-3E: biosecurity level-3 enhanced
CL: cloacal
dpc: day post-challenge
ECE: embryonating chickens eggs
EID_50_: mean egg infectious dose
HA: hemagglutinin
HE: hematoxylin-and-eosin
HI: hemagglutinin inhibition
HP: highly pathogenic
IHC: immunohistochemistry
LP: low pathogenic
MDT: mean death time
nd: not determined
OP: oropharyngeal
qRRT-PCR: quantitative real-time RT-PCR
SPF: specific pathogen free
USDA: United States Department of Agriculture
USNPRC: U.S. National Poultry Research Center
