# Supplementary material for: Pathobiology of Tennessee 2017 H7N9 low and high pathogenicity avian influenza viruses in commercial broiler breeders and specific pathogen free layer chickens
Source: Vet Res. 2018 Aug 29;49:82. doi: 10.1186/s13567-018-0576-0 (PMC6116495; doi:10.1186/s13567-018-0576-0)
Supplement: Supplementary file 1 — Additional file 1. Microscopic lesions and viral antigen distribution in tissues from chickens inoculated with H7N9 HPAI virus. Four week-old specific pathogen free (SPF) White Leghorn chickens were challenged with A/chicken/Tennessee/17-007147-2/2017 H7N9 HPAI virus and sampled at 2 dpc. [file 13567_2018_576_MOESM1_ESM.docx]

**Additional file 1 Microscopic lesions and viral antigen distribution in tissues from chickens inoculated with H7N9 HPAI virus and sampled at 2 dpc.**

| Tissue | HE score^a,b^ | Lesions | IHC score^c^ | Cell types expressing virus antigen |
| --- | --- | --- | --- | --- |
| Nasal | +++/++ | Epithelial cell necrosis and desquamation, rhinitis, sinusitis, mononuclear cell infiltrate | +++/+ | Nasal epithelial cells, mononuclear cells |
| Trachea | +/+ | Focal necrosis with mild lymphoplasmacytic inflammatory infiltrate | +/+ | Pseudostratified epithelial cells, mononuclear cells |
| Lung | +++/+++ | Interstitial pneumonia with edema, congestion, necrosis, monocytic infiltrate | +++/+++ | Epithelium of air capillaries, mononuclear cells, necrotic debris |
| Comb | ++/++ | Edema, hemorrhages, necrosis | ++/++ | Vascular endothelial cells, mononuclear cells, necrotic debris, feather follicle epithelium |
| Eye lid | +/+ | Subcutaneous edema | +/+ | Vascular endothelial cells, mononuclear cells |
| Heart | +/+ | Focal necrosis of myocytes | +++/++ | Myocytes |
| Brain | +/+ | Neuronal necrosis, gliosis. Chromatolysis of Purkinge cell layer | ++/+ | Neurons, Purkinje cells, ependymal cells, glial cells, endothelial cells |
| Proventriculus | -/- | nd | -/- | nd |
| Intestine | +/+ | Lymphohistiocytic infiltration in submucosa. | +/+ | Epithelial cells, mononuclear cells in lymphoid associated tissue |
| Pancreas | +/+ | Mild degeneration of individual pancreatic acinar cells | ++/+ | Pancreatic acinar cells |
| Liver | +/++ | Focal necrosis with lymphoplasmacytic inflammatory infiltrate | +/++ | Kupffer cells, hepatocytes, endothelial cells, macrophages |
| Spleen | +++/+++ | Multifocal areas of necrosis, hemorrhages, lymphoid depletion, hyperplasia of macrophage-phagocytic cells | +++/+++ | Mononuclear cells |
| Thymus | +/+ | Focal necrosis, mild lymphocyte depletion, apoptotic lymphocytes | +/+ | Mononuclear cells |
| Cloacal bursa | +/+ | Lymphocyte necrosis and apoptosis. Lymphocyte depletion, phagocytic hyperplasia | +/+ | Epithelial cells, mononuclear cells |
| Kidney | +/- | Focal necrosis of tubular epithelium with lymphoplasmacytic inflammation | +/- | Tubular epithelial and glomerular cells |
| Ovaries | -/- | nd | +/+ | Tegument/interstitial tissue |
| Adrenal gland | +++/++ | Multifocal areas of necrosis with mononuclear inflammatory infiltrate | +++/++ | Corticotrophic and corticotropic cells |
| Skeletal Muscle | -/- | nd | +/+ | Myocytes |

nd: not determined

^a^ Tissues collected from 2 birds (bird 1/bird 2).

^b^ Histopathology score: HE, histologic lesions: - = no lesions; + = mild; ++ = moderate; +++ = severe.

^c^ IHC, immunohistochemical staining: - = no antigen staining; + = infrequent; ++ = common; +++ = widespread.
